# Cross-Sectional Study Evaluating the Role of Autonomic Nervous System Functional Diagnostics in Differentiating Post-Infectious Syndromes: Post-COVID Syndrome, Chronic Fatigue Syndrome, and Lyme Disease

**DOI:** 10.3390/biomedicines13020356

**Published:** 2025-02-04

**Authors:** Branislav Milovanovic, Nikola Markovic, Masa Petrovic, Vasko Zugic, Milijana Ostojic, Milovan Bojic

**Affiliations:** 1 Institute for Cardiovascular Diseases “Dedinje”, 11000 Belgrade, Serbia; 2 Faculty of Medicine, University of Belgrade, 11000 Belgrade, Serbia

**Keywords:** post-infectious syndromes, autonomic nervous system dysfunction, Post-COVID syndrome, chronic fatigue syndrome, neurocardiology

## Abstract

**Background/Objectives**: Post-infectious syndromes, including Post-COVID syndrome, Chronic Fatigue Syndrome, and late-stage Lyme disease, are associated with overlapping clinical features and autonomic dysfunction. Despite shared symptoms such as fatigue and orthostatic intolerance, the underlying pathophysiology and specific patterns of autonomic dysfunction may differ. This study aimed to evaluate and compare autonomic nervous system function in these syndromes using multiple diagnostic modalities to identify unique characteristics and improve differentiation between these conditions. **Methods**: This cross-sectional study included 758 patients, which were divided into four groups: Post-COVID syndrome, Chronic Fatigue Syndrome following Post-COVID syndrome, Chronic Fatigue Syndrome unrelated to COVID-19, and late-stage Lyme disease. Autonomic nervous system function was assessed using cardiovascular reflex tests, the Head-Up Tilt Test, beat-to-beat analysis, five-minute electrocardiogram recordings, 24 h Holter electrocardiogram monitoring, and 24 h ambulatory blood pressure monitoring. Statistical analyses compared parameters across the groups, focusing on patterns of sympathetic and parasympathetic dysfunction. **Results**: The patients with Lyme disease showed distinct autonomic patterns, including a higher prevalence of orthostatic hypotension (53.4%) and changes in heart rate variability during the Head-Up Tilt Test suggestive of adrenergic failure. Compared to the other groups, patients with Lyme disease exhibited reduced baroreceptor sensitivity and diminished changes in frequency domain heart rate variability parameters during orthostatic stress. Parasympathetic dysfunction was less prevalent in the Lyme disease group, while the Post-COVID syndrome and Chronic Fatigue Syndrome groups showed more pronounced autonomic imbalances. **Conclusions**: The patients with Post-COVID syndrome, Chronic Fatigue Syndrome, and late-stage Lyme disease exhibited varying degrees and types of autonomic dysfunction. Late-stage Lyme disease is characterized by adrenergic failure and distinct hemodynamic responses, differentiating it from other syndromes. The functional assessment of autonomic nervous system function could aid in understanding and managing these conditions, offering insights for targeted therapeutic interventions.

## 1. Introduction

Post-infectious syndromes are a significant focus of interest in contemporary medicine and public discourse. Among these, Post-COVID syndrome has garnered particular attention due to the vast number of individuals recovering from SARS-CoV-2 infection. There is sustained interest in ‘long COVID’ syndrome both within the professional and general public audience with an overall aim of better understanding and solution to the problem [1]. However, a clear, standardized definition of Post-COVID syndrome remains elusive. Before major societies, as well as the World Health Organization (WHO), defined Post-COVID syndrome as the continuation or development of new symptoms three months after the initial SARS-CoV-2 infection, with these symptoms lasting for at least two months without other explanation, one of the definitions was the persistence of symptoms and signs 4 weeks after the resolution of acute state [2,3,4].

In contrast, Chronic Fatigue Syndrome (CFS) (also known as Myalgic Encephalomyelitis (ME)), (ME/CFS) is a chronic, multisystem, and debilitating condition that has been formally recognized in the International Classification of Diseases since 1969. Both syndromes share a remarkably similar clinical presentation, including chronic fatigue, post-exertional malaise—a hallmark of ME/CFS—cognitive impairment, orthostatic intolerance, and unrefreshing sleep [5]. Following the COVID-19 pandemic, the prevalence of ME/CFS has surged, particularly among individuals recovering from SARS-CoV-2 infection, with estimates suggesting that 10% of such patients develop ME/CFS [6]. Similarly, increases in ME/CFS were noted following the H1N1 pandemic and outbreaks of severe acute respiratory syndrome (SARS) and Middle East respiratory syndrome (MERS), with 27.1% of SARS survivors meeting the diagnostic criteria for ME/CFS [6].

Lyme disease, caused by the bacterium *Borrelia burgdorferi* and its various genospecies, presents another clinical condition with overlapping features. Differences in the causative genospecies result in variable antigenic presentations, clinical manifestations, and responses to antibiotic therapy [7,8]. While the acute phase of Lyme disease is often marked by erythema migrans, misdiagnosis can lead to delayed treatment. The late stage of Lyme disease is characterized by polymorphic symptoms, such as chronic fatigue, orthostatic intolerance, cognitive dysfunction, and migraines, which closely resemble the clinical features of Post-COVID syndrome and ME/CFS [9,10].

Emerging evidence suggests that immune dysregulation, pathogen-triggered autoimmunity, diminished natural killer cell and T lymphocyte function, neuroinflammation, and autonomic nervous system dysfunction are central to the pathogenesis of these syndromes [11,12,13,14]. Dysautonomia has been proposed as a critical component in Post-Treatment Lyme Disease Syndrome by Adler et al., while Słomko et al. emphasize the role of autonomic dysfunction phenotypes in patients with ME/CFS [11,14]. Symptoms such as orthostatic intolerance, postural tachycardia syndrome, and orthostatic hypotension are thought to result directly from autonomic dysfunction. The autonomic nervous system also plays a key role in modulating the immune system, particularly through the anti-inflammatory actions of the parasympathetic nervous system [15].

The primary aims of this study were to assess autonomic nervous system dysfunction in patients with Post-COVID syndrome, ME/CFS, and chronic Lyme disease, in order to provide evidence of an organic substrate in these syndromes. Additionally, this study aims to delineate distinct phenotypes of autonomic dysfunction across these post-infectious syndromes. Another aim was to explore potential differential diagnostic markers for these syndromes using functional diagnostics of the autonomic nervous system.

## 2. Materials and Methods

This cross-sectional study included 1129 patients aged 18 years and older who were examined at the Neurocardiological Laboratory of the Cardiology Clinic at the Institute for Cardiovascular Diseases “Dedinje”. Patients were divided into four groups based on specific inclusion criteria.

The first group, referred to as the Post-COVID syndrome group, consisted of 97 patients (mean age 46.54 ± 13.5 years; 40 males, 57 females) who fulfilled criteria for Post-COVID according to WHO [4]. The second group included 287 patients (mean age 46.3 ± 13.3 years; 88 males, 199 females) diagnosed with ME/CFS that developed after Post-COVID syndrome. The third group comprised 508 patients (mean age 43.98 ± 12.83 years; 165 males, 343 females) diagnosed with ME/CFS unrelated to SARS-CoV-2 infection, primarily following the 2021 criteria of the National Institute for Health and Care Excellence [16]. The fourth group, referred to as the Lyme disease group, included 237 patients (mean age 43.61 ± 14.46 years; 78 males, 159 females) with polymorphic complaints lasting longer than six months and confirmed through a two-tier serology protocol, which required positive results for both enzyme-linked immunosorbent assay or enzyme immunoassay and Western blot testing for the same antibody class (IgM or IgG) (Table 1).

Inclusion criteria for the respective groups were based on the clinical presentations and diagnostic protocols described above. Exclusion criteria for all groups included chronic diseases such as neurological, endocrinological, or autoimmune conditions that could explain the symptoms. Patients with neurological disorders causing primary autonomic failure, such as Pure Autonomic Failure, Multiple System Atrophy, or Parkinson’s disease, were also excluded. Most participants underwent thorough evaluations by specialists, including neurologists, rheumatologists, and endocrinologists, often on multiple occasions, to rule out diseases within their respective fields of expertise

The study was conducted according to the guidelines of the Declaration of Helsinki and approved by the Institutional Ethics Committee of Institute for Cardiovascular Diseases “Dedinje” (Number 6472 approved on 11 December 2024). This study was also supported by grant 451-03-68/2020-14/200156 from the Ministry of Education, Science, and Technological Development of the Republic of Serbia and by the COVANSA grant from the Science Fund of the Republic of Serbia. This study was performed in line with the principles of the Declaration of Helsinki.

### 2.1. Study Protocol

All patients underwent a comprehensive assessment of autonomic nervous system function using the following diagnostic modalities: cardiovascular reflex tests (CARTs) by Ewing, the Head-Up Tilt Test, beat-to-beat analysis using the Task Force Monitor, five-minute electrocardiogram recordings in the supine position and during the passive phase of the Head-Up Tilt Test, 24 h Holter electrocardiogram monitoring, and 24 h ambulatory blood pressure monitoring. A total of 1126 results from cardiovascular reflex tests, 550 five-minute electrocardiogram recordings, 477 beat-to-beat recordings, 392 complete 24 h Holter recordings, and 327 complete 24 h ambulatory blood pressure recordings were analyzed (Supplement S1).

#### 2.1.1. Cardiovascular Reflex Tests (CARTs)

The Ewing battery of cardiovascular reflex tests was used to evaluate autonomic nervous system function [17]. Sympathetic function was assessed using the Hand Grip Test and Blood Response to Standing, while parasympathetic function was evaluated using the Valsalva maneuver, Heart Response to Deep Breathing, and Heart Response to Standing. Test results were categorized as normal, borderline, or abnormal, and assigned numerical values (normal = 0, borderline = 1, abnormal = 2) [18]. These scores were summed to yield a total score ranging from 0 to 10. Sympathetic or parasympathetic dysfunction was defined by the presence of one or more abnormal tests. Complete autonomic dysfunction was characterized by concurrent sympathetic and parasympathetic dysfunction.

#### 2.1.2. Head-Up Tilt Test (HUTT)

The Head-Up Tilt Test (HUTT) was conducted following the Westminster protocol [19]. Patients were positioned supine for 10 min before being tilted to a 70° angle for up to 30 min. Continuous blood pressure and 12-lead ECG monitoring were performed. The test was deemed positive if syncope or severe presyncope occurred.

#### 2.1.3. Beat-to-Beat Analysis

Beat-to-beat analysis was performed using the Task Force Monitor to measure systolic blood pressure, diastolic blood pressure, mean arterial pressure, and heart rate [20]. Baroreceptor reflex sensitivity and baroreceptor effectiveness index were assessed using the sequence technique, and Δ values were calculated as the difference between parameters during the passive phase and supine phase of the Head-Up Tilt Test [21,22].

#### 2.1.4. Five-Minute ECG Recordings

Twelve-lead ECG recordings were conducted using a Cardioscan system, with five-minute recordings taken in the supine position and during the passive phase of the Head-Up Tilt Test. Parameters including P wave duration, PQ interval, QRS duration, QT interval, QTc interval, and T wave duration were analyzed. Spectral analysis of heart rate variability included components such as total power, very low frequency, low frequency, high frequency, and the low frequency to high frequency ratio.

#### 2.1.5. 24 h Holter ECG Monitoring

Twenty-four-hour Holter ECG monitoring was conducted to evaluate time and frequency domains of heart rate variability, QT and QTc intervals, deceleration and acceleration capacities, and heart rate turbulence parameters. Rhythm analysis included the total number of premature ventricular and atrial contractions.

#### 2.1.6. 24 h Ambulatory Blood Pressure Monitoring

Ambulatory blood pressure monitoring was performed using an oscillometric method to record systolic, diastolic, and mean arterial pressures as well as pulse pressure every 15 min over 24 h. Daytime (8 AM–10 PM) and nighttime (10 PM–8 AM) averages and their standard deviations were calculated.

### 2.2. Statistical Analysis

Results are presented as means with 95% confidence intervals or counts and percentages. Δ values were shown as means. Normality was assessed using the Smirnov test and Q–Q plots. Non-normally distributed data were log- or square-root-transformed for statistical analysis, with back-transformation performed for presentation. Parametric data were compared using analysis of variance with Tukey’s honestly significant difference post hoc tests, while nonparametric data were analyzed using Chi Square and Mann–Whitney U tests. Statistical analyses were conducted using SPSS software version SPSS 29.0 (IBM Corp. Released 2022. IBM SPSS Statistics for Windows, Version 29.0. Armonk, NY, USA: IBM Corp.) with significance set at *p* < 0.05.

## 3. Results

### 3.1. Cardiovascular Reflex Tests (CARTs)

Abnormal results for the Hand Grip Test were less frequent in the Lyme disease group compared to the other three groups (*p* < 0.05). Conversely, the patients with Lyme disease exhibited a significantly higher proportion of borderline and abnormal results for the Orthostatic Hypotension Test compared to the other groups (*p* < 0.05).

The Valsalva maneuver showed more frequent abnormal results in the patients with ME/CFS following Post-COVID syndrome (Group 2) than in the other groups (*p* < 0.05). For the Heart Response to Standing Test, abnormal results were less common in the Lyme disease group (*p* < 0.05). Overall, complete autonomic dysfunction was less prevalent in the patients with Lyme disease than in the other groups (*p* < 0.05) (Table 2).

### 3.2. Beat-to-Beat Analysis

**Figure 1:**
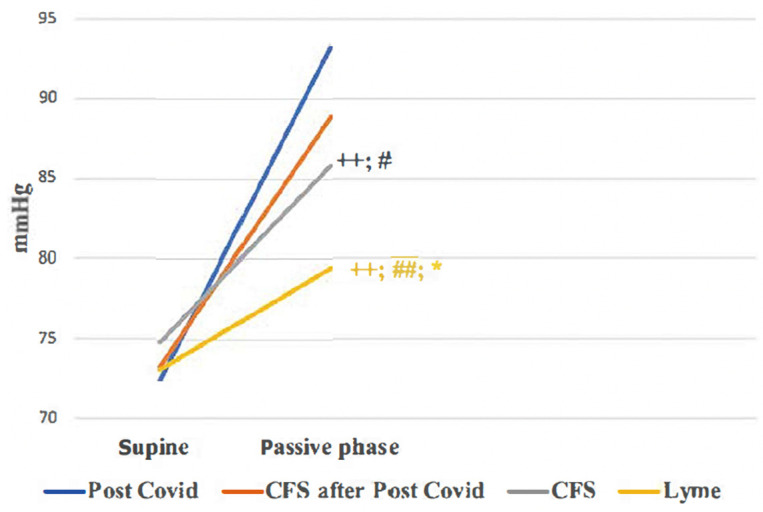
Changes in diastolic blood pressure during HUTT. +—*p* value for comparing ΔDBP of 1st group with ΔDBP of groups 2, 3, and 4 (+—*p* < 0.05; ++—*p* < 0.01); #—*p* value for comparing ΔDBP 2nd group with ΔDBP of group 3 and group 4 (#—*p* < 0.05; ##—*p* < 0.01); *—*p* value for comparing ΔDBP of the 3rd group with ΔDBP of the 4th group (*—*p* < 0.05; **—*p* < 0.01); all *p*-values were adjusted for pairwise comparisons using the Bonferroni correction.

ΔDiastolic blood pressure during the HUTT showed significant differences between all groups (*p* < 0.05), except between the Post-COVID (Group 1) and Post-COVID ME/CFS (Group 2) groups (Table 3 and Figure 1). The lowest Δdiastolic blood pressure was observed in the Lyme disease group, while the highest was noted in the Post-COVID group.

**Figure 2:**
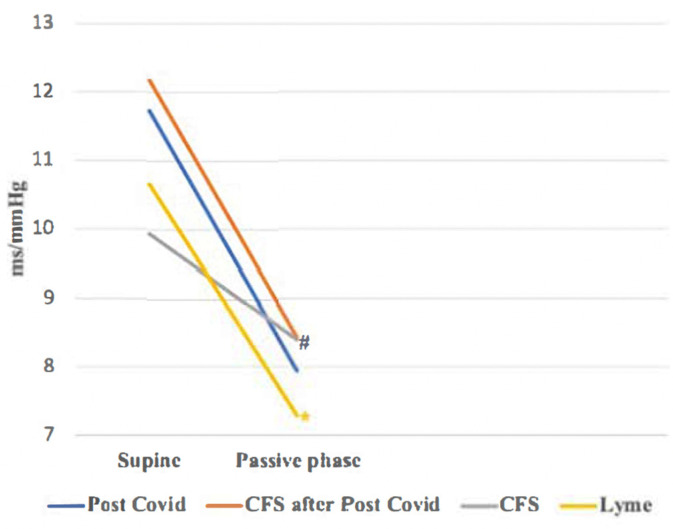
Changes in baroreflex sensitivity during HUTT. #—*p* value for comparing ΔBRS 2nd group with ΔBRS of groups 3 and 4 (#—*p* < 0.05; ##—*p* < 0.01); *—*p* value for comparing ΔBRS of 3rd group with ΔBRS of 4th group (*—*p* < 0.05; **—*p* < 0.01); all *p*-values were adjusted for pairwise comparisons using the Bonferroni correction.

The baroreflex effectiveness index during the passive phase of the HUTT was significantly higher in the Lyme disease group compared to the other groups (*p* < 0.001). Baroreflex sensitivity decreased during the Head-Up Tilt Test across all groups, with CFS unrelated to COVID-19 (Group 3) showing a lesser decrease compared to the Post-COVID (Group 1, *p* > 0.05), Post-COVID CFS (Group 2, *p* < 0.05), and Lyme disease groups (Group 4, *p* < 0.05) (Table 2 and Figure 2).

### 3.3. Five-Minute ECG Recording

The Lyme disease group exhibited the lowest heart rate and QTc duration during the passive phase of the HUTT. Heart rate in the Lyme disease group was significantly lower compared to the Chronic Fatigue Syndrome group unrelated to COVID-19 (Group 3, *p* < 0.05), while the QTc duration was significantly shorter compared to the Chronic Fatigue Syndrome group following Post-COVID syndrome (Group 2, *p* < 0.05) (Table 4).

**Figure 3:**
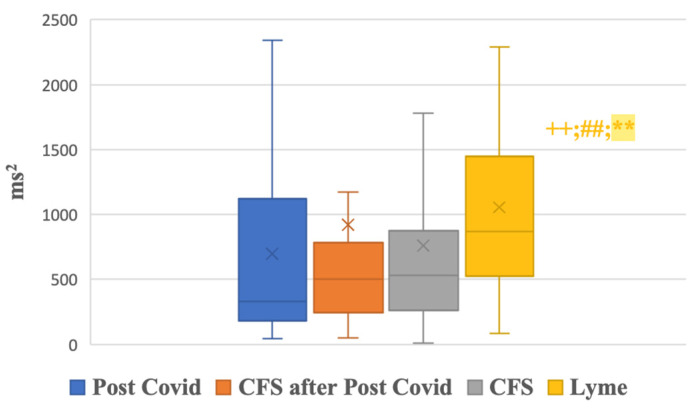
VLF (ms^2^) in supine position. VLF—very-low-frequency component of HRV; +—*p* value for comparing the 1st group with groups 2, 3, and 4 (+—*p* < 0.05; ++—*p* < 0.01); #—*p* value for comparing the 2nd group with groups 3 and 4 (#—*p* < 0.05; ##—*p* < 0.01); *—*p* value for comparing the 3rd group with the 4th group (*—*p* < 0.05; **—*p* < 0.01); all *p*-values were adjusted for pairwise comparisons using the Bonferroni correction.

In the supine position, the total power of the heart rate variability was highest in the Lyme disease group, with a statistically significant difference when compared to the Chronic Fatigue Syndrome group following Post-COVID syndrome (Group 2, *p* < 0.01). The very-low-frequency (VLF) heart rate variability was significantly higher in the Lyme disease group compared to all the other groups (*p* < 0.05) (Figure 3).

Low frequency (LF) was lowest in Group 2, with statistically significant differences when compared to Group 3 and the Lyme disease group (*p* < 0.05) (Table 5).

During the passive phase of HUTT, the Lyme disease group demonstrated lower values for SDNN, rMSSD, SDSD, total power, VLF, LF/HF (both in ms^2^ and normalized units), and LFnu compared to the other groups. However, only SDSD, rMSSD, and pNN50 were significantly lower in the Lyme disease group when compared to the ME/CFS group unrelated to COVID-19 (Group 3, *p* < 0.05) (Table 6).

**Figure 4:**
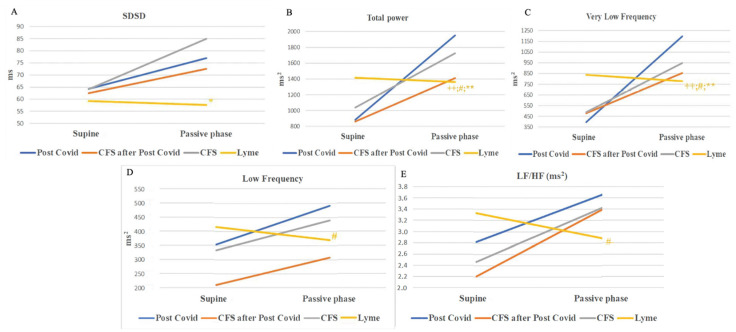
(**A**) Changes in SDSD, (**B**) total power, (**C**) very low frequency, (**D**) low frequency, (**E**) LF/HF (expressed in ms^2^) during HUTT between the groups. SDSD—the standard deviation of the successive differences between adjacent normal RR interval; total power of HRV; very-low-frequency component of HRV (0–0.05 Hz); low-frequency component of HRV (0.05–0.17 Hz); LF/HF—low-frequency/high-frequency ratio of HRV; +—*p* value for comparing Δ values of the 1st group with groups 2, 3, and 4 (+—*p* < 0.05; ++—*p* < 0.01); #—*p* value for comparing Δ values of 2nd group with groups 3 and 4 (#—*p* < 0.05; ##—*p* < 0.01); *—*p* value for comparing Δ values of the 3rd group with the 4th group (*—*p* < 0.05; **—*p* < 0.01); all *p*-values were adjusted for pairwise comparisons using the Bonferroni correction.

A unique pattern was observed in the Lyme disease group during HUTT, where the SDSD, total power, VLF, LF (ms^2^), and LF/HF (ms^2^) decreased, with the ΔTP and ΔVLF showing statistically significant differences compared to the other groups (*p* < 0.05) (Figure 4).

**Figure 5:**
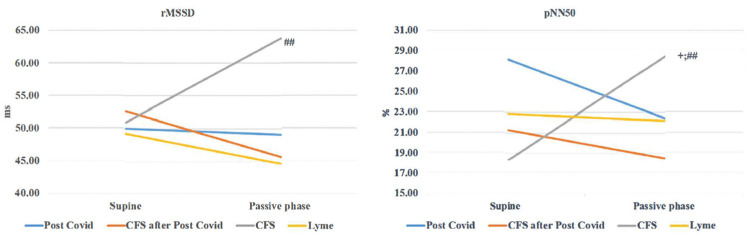
Changes in rMSSD and pNN50 during HUTT. rMSSD—the square root of the mean of the squares of the successive differences between adjacent normal RR intervals; pNN50—the percentage of intervals > 50 ms different from preceding interval; +—*p* value for comparing the 1st group with groups 2, 3, and 4 (+—*p* < 0.05; ++—*p* <0.01); #—*p* value for comparing the 2nd group with groups 3 and 4 (#—*p* < 0.05; ##—*p* < 0.01); *—*p* value for comparing the 3rd group with the 4th group (*—*p* < 0.05; **—*p* < 0.01).

Conversely, in the Chronic Fatigue Syndrome group unrelated to COVID-19 (Group 3), rMSSD and pNN50 decreased during the passive phase, with some statistical differences in the Δ values when compared to the other groups (*p* < 0.05) (Figure 5).

### 3.4. 24 h Holter ECG Monitoring

No statistically significant differences were observed between the groups during 24 h Holter ECG monitoring.

### 3.5. 24 h Ambulatory Blood Pressure Monitoring

Systolic and diastolic blood pressures during nighttime were significantly lower in the Lyme disease group (systolic: 111.4 ± 14.3; diastolic: 66.7 ± 9.2) compared to the ME/CFS group (systolic: 118.2 ± 16.6; diastolic: 70.6 ± 10.8) (Group 3, *p* < 0.05). The overall diastolic blood pressure was significantly lower in the Lyme disease group (73.0 ± 8.4) than in the Post-COVID ME/CFS group (77.1 ± 9.1) (Group 2, *p* < 0.05).

## 4. Discussion

Dysautonomia has long been recognized as a pathological phenomenon in patients with Post-COVID syndrome and ME/CFS, but its occurrence in Lyme disease has received less attention. This study aimed to address this gap, using autonomic nervous system testing to assess dysfunction across these post-infectious syndromes.

Abnormal Hand Grip Test results were statistically less frequent in the Lyme disease group, potentially underestimating the prevalence of sympathetic dysfunction in these patients based on Ewing’s criteria. The utility of the Hand Grip Test in the diagnostic battery has been debated due to its dependence on the baseline systolic pressure and its weak correlation with other autonomic tests [23]. Conversely, orthostatic hypotension, a major marker of sympathetic dysfunction, was significantly more prevalent in the Lyme disease group, occurring in 53.4% of the patients. This underscores the need to carefully evaluate the inclusion of Hand Grip Test results in defining sympathetic dysfunction.

Parasympathetic dysfunction was less common in the Lyme disease group than in the other groups, as evidenced by the lower abnormal results in the Heart Response to Breathing, Heart Response to Standing, and Valsalva maneuver tests [24,25]. These findings align with the reduced prevalence of definite parasympathetic dysfunction observed in Lyme disease patients. Previous studies have similarly highlighted autonomic dysfunction in post-infectious syndromes, including Post-COVID and ME/CFS. For instance, Salem et al. and Yar et al. reported higher rates of sympathetic dysfunction in Post-COVID syndrome compared to healthy populations [26,27]. In Lyme disease, Novak et al. identified sympathetic and parasympathetic dysfunction in patients with Post-Treatment Lyme Disease Syndrome [28].

The HUTT revealed distinct patterns of autonomic dysfunction. In ME/CFS, markers of parasympathetic activity, such as rMSSD and pNN50, increased during the initial five minutes of HUTT, suggesting a compensatory mechanism to orthostatic stress. In contrast, these markers decreased in the Lyme disease group, accompanied by reductions in the total power, very low frequency (VLF), low frequency (LF), and the LF/HF ratio (expressed in ms^2^). These findings suggest efferent adrenergic failure, impairing blood pressure control during orthostatic stress. Additionally, the Lyme disease group demonstrated diminished changes in heart rate and baroreflex sensitivity (BRS), further supporting the presence of adrenergic dysfunction.

The frequency domain analysis of heart rate variability (HRV) showed higher resting values of VLF and total power in the Lyme disease group compared to the other groups. VLF is associated with various physiological processes, including inflammation, thermoregulation, and the renin–angiotensin–aldosterone system (RAAS) [29,30,31,32,33,34]. However, the LF/HF ratio, indicative of the sympathetic–vagal balance, exceeded 2 in all groups, reflecting sympathetic dominance. This finding aligns with studies suggesting parasympathetic dysfunction in Post-COVID syndrome and ME/CFS [26,27,35].

During 24 h ambulatory blood pressure monitoring, the Lyme disease group exhibited a significantly lower nighttime systolic and diastolic blood pressure compared to the other groups. This finding, along with the high prevalence of orthostatic hypotension, suggests that patients with Lyme disease may benefit from pharmacological interventions, such as midodrine, to support blood pressure regulation. This aligns with Newton et al.’s findings in ME/CFS patients, who reported lower blood pressure and proposed sympathomimetic treatments for such phenotypes [36].

The observed autonomic dysfunction in all the groups highlights the complex interplay between the autonomic and immune systems in post-infectious syndromes. Dysautonomia can explain the overlapping symptoms such as fatigue, orthostatic intolerance, and sleep disturbances. However, discrepancies between the objective measures of autonomic dysfunction and subjective symptoms, as noted in prior studies, emphasize the need for further investigation [37].

Mechanistically, autonomic instability may arise from immune-mediated damage or direct microbial invasion of the autonomic nervous system. Koralnik and Tyler proposed that viral invasion, inflammatory responses, and prothrombotic states contribute to neurological and autonomic manifestations of COVID-19 [38]. Similarly, Durray’s findings of inflammatory infiltrates in the autonomic ganglia of Lyme disease patients suggest a pathogenic role for immune dysfunction [39,40]. The bidirectional interaction between the immune and autonomic systems underscores the need for integrative approaches to understanding and managing these syndromes [40].

It is important to note that public discourse and awareness have been central to the evolution of long COVID’s recognition, as exemplified by a recent study analyzing over 1.3 million tweets, which highlighted the sustained public interest and community support around this condition [1]. The study brought to light the multifaceted impact of long COVID, ranging from neurocognitive deficits and chronic fatigue to economic and labor implications. These narratives demonstrate the ongoing need for research and targeted interventions to mitigate the long-term impacts of this syndrome and support those affected.

### Study Limitations

This study has several limitations. The groups were not matched for age or gender, and there was a disproportionate number of patients in each group. Additionally, not all participants underwent every diagnostic modality, which may have introduced bias. These limitations should be considered when interpreting the findings, and future studies should aim for more balanced and comprehensive datasets. Regarding the nature of this syndromes, we fully acknowledge the inherent challenges posed by the prodromal phases and variable courses of certain neurological diseases, which can complicate the diagnostic process for these post-infectious syndromes, and also the need for patients being evaluated by other specialists (neurologists, rheumatologists, etc.). The aforementioned state also represents a study limitation. It is important to emphasize for further studies the use of recommended criteria for the diagnosis of this syndrome proposed by representative institutions.

## 5. Conclusions

This study highlights the presence of autonomic dysfunction in patients with post-infectious syndromes, including Post-COVID syndrome, ME/CFS, and chronic Lyme disease. Using various diagnostic modalities, suggestive trends of autonomic dysfunction were identified across these conditions. Notably, the patients with chronic Lyme disease demonstrated signs of efferent adrenergic failure, as evidenced by changes observed during HUTT and the high prevalence of orthostatic hypotension (OH) in over 50% of the cases. The results suggest that functional diagnostic assessments of the autonomic nervous system can provide valuable insights into the pathophysiological mechanisms underlying these syndromes. Further research is warranted to refine these diagnostic approaches and explore their clinical applications.

## Figures and Tables

**Table 1:** Study cohort demographic characteristics by group.

	Post-COVID (n = 97)	ME/CFS After Post-COVID (n = 284)	ME/CFS (n = 508)	Lyme (n = 237)	*p* Value
Male (n,%)	40 (41.2%)	87 (30.5%)	165 (32.5%)	78 (32.9%)	0.281 ^a^
Female (n,%)	57 (58.8%)	198 (69.5%)	343 (67.5%)	159 (67.1%)	
Age	46.5 ± 13.5	46.3 ± 13.3	44 ± 12.8	43.6 ± 14.5	0.034 ^b^

ME/CFS—Myalgic Encephalomyelitis/Chronic Fatigue Syndrome; ^a^—Pearson’s Chi Square; ^b^—One-way Anova.

**Table 2:** Results of CARTs among the groups.

	Post-COVID (n = 97)	ME/CFS After Post-COVID (n = 284)	ME/CFS (n = 508)	Lyme Disease (n = 237)
HGT (n,%)	94 (96.9%)	269 (94.7%)	489 (96.3%)	206 (86.9%) ^+;##;^**
OH (borderline) (n,%)	4 (4.1%)	24 (8.5%)	35 (6.9%)	91 (38.6%) ^++;##;^**
OH (abnormal) (n,%)	11 (11.3%)	33 (11.6%)	69 (13.6%)	35 (14.8%) ^++;##;^**
DS (n,%)	94 (96.9%)	269 (94.7%)	490 (96.5%)	210 (89%) ^+;#;^**
VM (abnormal) (n,%)	25 (25.8%)	101 (35.6%) ^+^	136 (26.8%) ^#^	57 (24.3%) ^##^
HRB (n,%)	66 (68%)	207 (72.9%)	365 (72%)	144 (61.3%) ^##;^**
HRS (n,%)	89 (91.8%)	254 (89.4%)	462 (91.1%)	202 (86%) ^+;#;^*
Definite DP (n,%)	72 (74.2%)	221 (77.8%)	367 (72.4%)	147 (62.6%) ^#;^**
CAD (n,%)	92 (94.8%)	264 (93%)	479 (94.5%)	206 (87.1%) ^+;#;^**
Score	6.44 (6.17–6.72)	6.61 (6.45–6.77)	6.52 (6.39–6.65)	6.43 (6.22–6.64)

For HGT, VM, HRB, and HRS, only abnormal results of tests are shown in Table 2. CARTs—cardiovascular reflex tests; HGT—Hand Grip Test; OH—Orthostatic Hypotension Test (blood pressure response to standing); DS—sympathetic dysfunction; VM—Heart Response to Valsalva maneuver; HRB—Heart Response to breathing; HRS—Heart Response to standing; DP—parasympathetic dysfunction; CAD—complete autonomic dysfunction; +—*p* value for comparing 1st group with groups 2, 3, and 4 (+—*p* < 0.05; ++—*p* < 0.01); #—*p* value for comparing 2nd group with groups 3 and 4 (#—*p* < 0.05; ##—*p* < 0.01); *—*p* value for comparing 3rd group with the 4th group (*—*p* < 0.05; **—*p* < 0.01); all *p*-values were adjusted for pairwise comparisons using the Bonferroni correction.

**Table 3:** Beat-to-beat parameters.

Parameters	Post-COVID (n = 35)	ME/CFS After Post-COVID (n = 127)	ME/CFS (n = 186)	Lyme Disease (n = 129)
SBP (supine) (mmHg)	115.02 (110.17–119.87)	114.89 (112.05–117.72)	117.12 (114.51–111.89)	108.84 (103.12–114.57)
SBP (pasisve phase) (mmHg)	132.09 (124.87–139.31)	127.79 (124.31–131.27)	125.25 (122.2–128.3)	123.71 (117.10–130.32)
DBP (supine) (mmHg)	72.51 (68.61–76.42)	73.35 (71.19–75.51)	74.85 (72.69–77.01)	73.18 (71.04–75.32)
DBP (passive phase) (mmHg)	93.29 (87.36–99.21)	88.88 (85.98–91.77)	85.83 (83.09–88.57)	79.49 ^++;##;^** (76.87–82.11)
BRS (supine) (ms/mmHg)	11.73 (9.01–15.29)	12.17 (10.90–13.59)	9.94 ^#^ (9.03–10.94)	10.66 (9.51–11.95)
BRS (passive phase) (ms/mmHg)	7.94 (6.26–10.07)	8.42 (7.55–9.40)	8.39 (7.59–9.27)	7.29 (6.43–8.28)
BEI (supine) (%)	67.35 (58.47–76.23)	66.37 (62.67–70.08)	60.42 (56.42–64.41)	85.36 ^##;^** (75.53–95.19)
BEI (passive phase) (%)	64.41 (57.01–71.82)	62.71 (58.74–66.69)	60.72 (55.96–65.48)	88.26 ^++;##;^** (78.67–97.86)

SBP—systolic blood pressure; DBP—diastolic blood pressure; BRS—baroreflex sensitivity; BEI—baroreflex effectiveness index; +—*p* value for comparing 1st group with groups 2, 3, and 4 (+—*p* < 0.05; ++—*p* < 0.01); #—*p* value for comparing 2nd group with groups 3 and 4 (#—*p* < 0.05; ##—*p* < 0.01); *—*p* value for comparing 3rd group with 4th group (*—*p* < 0.05; **—*p* < 0.01); all p-values were adjusted for pairwise comparisons using the Bonferroni correction.

**Table 4:** ECG parameters during five minutes’ recording.

Parameters	Post-COVID (n = 42)	ME/CFS After Post-COVID (n = 134)	ME/CFS (n = 286)	Lyme (n = 88)
Heart rate (supine) (bpm)	76.62 (72.28–80.96)	76.25 (73.83–78.68)	77.10 (75.55–78.66)	73.34 (70.47–76.21)
Heart rate (passive phase) (bpm)	78.36 (74.42–82.29)	78.07 (75.66–80.49)	78.78 (77.15––80.41)	73.55 * (70.98–76.1)
P wave duration (supine) (ms)	101.43 (93.76–109.1)	94.13 (91.26–96.99)	94.8 (92.48–97.12)	95.77 (92.08–99.46)
P wave duration (passive phase) (ms)	93.88 (87.72–100.04)	91.50 (88.69–94.31)	93.32 (91.36–95.28)	97.62 ^#^ (93.77–101.48)
ΔPQ (ms)	−16	−2.96	−0.65 ^+^	−0.9
QTc duration (supine) (ms)	441.14 (430.79–451.49)	429.18 (420.62––437.73)	429.46 (425.1–433.82)	425.77 (419.10–432.44)
QTc duration (passive phase (ms)	437.67 (428.76–446.58)	439.37 (430.83–447.91)	429.27 (424.97–433.57)	424.28 ^#^ (415.64–432.92)

+—*p* value for comparing the 1st group with groups 2, 3, and 4 (+—*p* < 0.05; ++—*p* < 0.01); #—*p* value for comparing the 2nd group with groups 3 and 4 (#—*p* <0.05; ##—*p* < 0.01); *—*p* value for comparing 3rd group with 4th group (*—*p* < 0.05; **—*p* < 0.01); all p-values were adjusted for pairwise comparisons using the Bonferroni correction.

**Table 5:** Values of HRV parameters during supine position.

Parameters	Post-COVID (n = 42)	ME/CFS After Post-COVID (n = 134)	ME/CFS (n = 286)	Lyme (n = 88)
SDNN (ms)	64.30 (53.75–76.91)	64.46 (56.39–73.69)	64.82 (58.86–71.38)	58.99 (50.68–68.68)
rMSSD (ms)	50.06 (38.24–65.54)	52.70 (42.96–64.64)	50.96 (43.85–59.21)	39.40 (31.53–49.24)
SDSD (ms)	64.25 (53.72–76.86)	62.52 (54.43–71.81)	64.14 (55.71-73.84)	59.22 (51.13-68.6)
pNN50 (%)	28.16 (21.33–35.94)	21.31 (15.83–27.62)	18.35 (14.67–22.45)	22.87 (14.67–32.89)
TI (ms)	8.64 (7.24–10.31)	8.37 (7.66–9.14)	8.23 (7.81–8.68)	8.69 * (7.79–9.68)
TP (ms^2^)	884.50 (590.88–1324.04)	862.58 (703.40–1057.79)	1038.96 (909.91–1186.31)	1415.14 ^##^ (1170.85–1710.41)
VLF (ms^2^)	396.83 (248.31–634.16)	478.96 (372.31–616.17)	489.33 (419.95–570.16)	837.14 ^++;##;^** (692.63–1011.81)
LF (ms^2^)	355.04 (216.05–528.37)	210.64 (172.52–252.57)	334.12 ^#^ (286.35–385.57)	416.67 ^##^ (332.91–509.83)
HF (ms^2^)	153.57 (100–218.58)	150.88 (104.83–205.3)	188.04 (152.95–226.76)	197.01 (149.94–250.5)
LFnu (%)	63.59 (55.61–72.1)	60.1 (54.11–66.41)	63.76 (60.82–66.77)	65.68 (61.28–70.22)
HFnu (%)	32.08 (24.44–40.76)	34.96 (30.07–40.22)	32.54 (29.97–35.22)	30.94 (26.65–35.56)
LF/HF (ms^2^)	2.82 (1.69–4.25)	2.21 (1.74–2.74)	2.47 (2.17–2.78)	3.34 (2.55–4.22)
LF/HF (nu)	2.83 (1.69–4.26)	2.24 (1.76–2.79)	2.44 (2.14–2.76)	2.89 (2.13–3.76)

SDNN—standard deviation of normal RR intervals; rMSSD—the square root of the mean of the squares of the successive differences between adjacent normal RR intervals; SDSD—the standard deviation of the successive differences between adjacent normal RR interval; pNN50—the percentage of intervals > 50 ms different from preceding interval; TI—triangular index; TP—total power of HRV; VLF—very-low-frequency component of HRV (0–0.05 Hz); LF—low-frequency component of HRV (0.05–0.17 Hz); HF—high-frequency component of HRV (0.17–0.4 Hz); LF/HF—low-frequency/high-frequency ratio of HRV; nu—normalized units; +—*p* value for comparing the 1st group with groups 2, 3, and 4 (+—*p* < 0.05; ++—*p* < 0.01); #—*p* value for comparing the 2nd group with groups 3 and 4 (#—*p* <0.05; ##—*p* < 0.01); *—*p* value for comparing the 3rd group with the 4th group (*—*p* < 0.05; **—*p* < 0.01); all *p*-values were adjusted for pairwise comparisons using the Bonferroni correction.

**Table 6:** Values of HRV parameters during passive phase of Head-Up Tilt Test.

Parameters	Post-COVID (n = 42)	ME/CFS After Post-COVID (n = 134)	ME/CFS (n = 286)	Lyme (n = 88)
SDNN (ms)	76.98 (64.19–92.32)	74.20 (65.10–84.57)	88.41 (80.85–96.67)	64.82 (56.77–74.01)
rMSSD (ms)	49.11 (35.73–67.51)	45.73 (36.77–56.87)	63.77 (62.16–65.42)	36.29 (29.47–44.69)
SDSD (ms)	77.02 (64.21–92.38)	72.49 (63.26–83.08)	85 (77.52–93.20)	57.69 (49.82–66.80)
pNN50 (%)	22.38 (14.67–31.70)	18.51 (12.49–25.71)	28.57 (24.25–33.23)	22.18 (13.98–32.26)
TI (ms)	10.81 (9.22–12.68)	9.91 (9.13–10.76)	10.29 (9.71–10.91)	9.96 (9.13–10.86)
TP (ms^2^)	1949.40 (1409.94–2695.26)	1411.24 (1048.33–1899.77)	1723.06 (1048.33–2832.04)	1361.44 (1065.86–1739)
VLF (ms^2^)	1195.36 (831.96–1717.51)	854.87 (665.89–1097.49)	947.98 (808.91–1110.96)	776.96 (610.24–989.24)
LF (ms^2^)	491.40 (348.11–659.33)	308.66 (227.37–402.35)	440.06 (368.95–517.43)	370.42 (294.21–455.40)
HF (ms^2^)	190.74 (100.87–309)	135.67 (82.30–202.33)	172.66 (137.22–212.17)	167.05 (125.81–214.12)
LFnu (%)	72.40 (66.02–79.08)	70.03 (65.25–74.98)	70.39 (67.62–73.21)	67.49 (62.44–72.75)
HFnu (%)	24.81 (19.40–30.88)	26.06 (22.10–30.35)	26.15 (23.75–28.66)	28.43 (24.39–32.78)
LF/HF (ms^2^)	3.66 (2.52–5.01)	3.39 (2.61–4.28)	3.43 (2.94–3.95)	2.89 (2.22–3.66)
LF/HF (nu)	3.66 (2.53–5.01)	3.60 (2.82–4.48)	3.53 (3.06–4.03)	3.11 (2.43–3.89)

SDNN—standard deviation of normal RR intervals; rMSSD—the square root of the mean of the squares of the successive differences between adjacent normal RR intervals; SDSD—the standard deviation of the successive differences between adjacent normal RR interval; pNN50—the percentage of intervals > 50 ms different from preceding interval; TI—triangular index; TP—total power of HRV; VLF—very-low-frequency component of HRV (0–0.05 Hz); LF—low-frequency component of HRV (0.05–0.17 Hz); HF—high-frequency component of HRV (0.17–0.4 Hz); LF/HF—low-frequency/high-frequency ratio of HRV; nu—normalized units; +—*p* value for comparing 1st group with groups 2, 3, and 4 (+—*p* < 0.05; ++—*p* < 0.01); #—*p* value for comparing 2nd group with group 3 and 4 (#—*p* < 0.05; ##—*p* < 0.01); *—*p* value for comparing 3rd group with 4th group (*—*p* < 0.05; **—*p* < 0.01); all *p*-values were adjusted for pairwise comparisons using the Bonferroni correction.

## Data Availability

The data are available upon reasonable request.

## Supplementary Materials

The following supporting information can be downloaded at: https://www.mdpi.com/article/10.3390/biomedicines13020356/s1.
